# Emerging Trends in Research on Self-Regulated Learning and Implications for Education: An Introduction to the Special Issue

**DOI:** 10.3390/jintelligence11030052

**Published:** 2023-03-09

**Authors:** Sarah K. Tauber, Robert Ariel

**Affiliations:** 1 Department of Psychology, Texas Christian University, Fort Worth, TX 76129, USA; 2 Department of Psychology, Virginia Wesleyan University, Virginia Beach, VA 23455, USA

Students in higher education face a multitude of challenges when gaining and refining their knowledge. They are assigned educational activities to complete on their own, and they learn new information in preparation for class assessments. To succeed, students must effectively take charge of their learning to meet achievement goals and maintain knowledge over time. This often occurs outside of classes and with minimal supervision. Thus, it is critical that students make effective self-regulated study choices.

Self-regulated learning is a multifaceted construct that involves any self-initiated cognitive or behavioral activity used to achieve a learning goal. This includes goal setting, planning, judgment and decision making, and strategy utilization (Dunlosky and Ariel 2011; Morisano et al. 2010; Winne and Hadwin 1998; Zimmerman and Schunk 2001). The choices that students make about their learning can be influenced by metacognitive processing (Dunlosky and Rawson 2012; Metcalfe and Finn 2008; Thiede et al. 2003). Metacognition refers to knowledge about learning, and the interactive relationship between assessments of learning and control of study behavior. Essential metacognitive processes include knowledge about effective study strategies and accurate awareness of knowledge acquisition (for reviews, see Dunlosky and Tauber 2014, 2016). Because these interrelated metacognitive and self-regulated processes can directly and indirectly impact actual learning, they are the focus of this Special Issue.

This Special Issue was open to empirical and theoretical contributions to education and cognitive psychology that specifically increased understanding of self-regulated learning and metacognition. We welcomed basic research with implications for educational contexts and applied research adopting educational materials and considering classroom environments. Our goal was for the Special Issue to address key concepts vital to multiple fields and to highlight a variety of experimental practices that are effective for answering long-standing questions about students’ self-regulated learning and education.

Each paper in this Special Issue provides unique insight into aspects of students’ self-regulated learning and thought-provoking discussion of application to educational contexts. All papers merit dedicated attention. In combination, multiple themes emerge from this outstanding work. Many of the papers used a mixed-methods approach, which is an exciting direction for the future of the field (Babineau et al. 2022; Rea et al. 2022; Zepeda and Nokes-Malach 2023). Mixed-method approaches combining quantitative and qualitative measures provide rich information about self-regulated learning and more insight than can be gathered by using one form of measurement. In further consideration of measurement, the form and types of judgments that students make can be critical for drawing conclusions about the accuracy of students’ metacognitive processing (Hughes and Thomas 2022; Zepeda and Nokes-Malach 2023). Another clear theme was the focus on students’ beliefs and knowledge about their learning in contrast with their study choices (Babineau et al. 2022; Macaluso et al. 2022; Rea et al. 2022). In some cases, students were knowledgeable about effective strategies (Rea et al. 2022), but they also demonstrated gaps in their understanding (Macaluso et al. 2022), and in all cases, disconnects between their knowledge and strategy utilization arose (Babineau et al. 2022; Macaluso et al. 2022; Rea et al. 2022).

The research in this Special Issue can inform interventions for improving students’ self-regulated learning skills. Interventions should target strategy knowledge, metacognitive monitoring processes, and the perceived costs of strategy implementation. Students’ default study strategies can create a false sense of fluency during learning (Macaluso et al. 2022). We need to break students’ bad study habits. The first step for doing so is to build a knowledgeable student who understands the appropriate strategies for achieving their learning goals. The next step is to find ways to properly motivate students to use effortful strategies. Even a knowledgeable student will avoid using effective strategies when they perceive the costs in effort for strategy implementation are too high (Macaluso et al. 2022; Rea et al. 2022). Learners strive for efficiency during self-regulated learning (Ariel et al. 2009) and effortful strategy use can be in conflict with this goal. A final step is to ensure that students can accurately monitor their learning (Hughes and Thomas 2022; Zepeda and Nokes-Malach 2023). Students’ ideas about their learning are imperfect (cf. Finn and Tauber 2015; Carpenter et al. 2020), and a simple way to improve monitoring is for students to retrieve criterial information during learning. Doing this can increase their preference for using some effortful strategies such as interleaved practice (Babineau et al. 2022).

In conclusion, the contributions in this Special Issue increase our understanding of students’ study choices and metacognitive processes, and they bring new excitement to these fields. These issues are directly relevant for students’ learning, and our hope is that this Special Issue encourages research with educational materials, in classroom settings, and focused on application to educational contexts. Discovering methods to improve self-regulated processes remains critical, and the work in this Special Issue should serve as a foundation for developing interventions to support students’ self-regulated learning.
